# Community health volunteers as a frontline platform for antimicrobial resistance mitigation in sub-Saharan Africa: A scoping review

**DOI:** 10.1371/journal.pgph.0006640

**Published:** 2026-06-11

**Authors:** Briton M. Kavulavu, Eric O. Omwenga, Eden H. Bukosia, Norrah N. Muendo, Rael J. Too

**Affiliations:** 1 Centre for Microbiology Research, Kenya Medical Research Institute (KEMRI), Nairobi, Kenya; 2 Department of Medical Microbiology, School of Biomedical Sciences, Jomo Kenyatta University of Agriculture and Technology, Nairobi, Kenya; 3 School of Medicine, Moi University, Eldoret, Kenya; 4 Department of Medical Microbiology and Parasitology, School of Health Sciences, Kisii University, Kisii, Kenya; University of Oxford, UNITED KINGDOM OF GREAT BRITAIN AND NORTHERN IRELAND

## Abstract

Antimicrobial resistance (AMR) is a global public health crisis disproportionately affecting sub-Saharan Africa (SSA), where it was directly responsible for approximately 255,000 deaths in 2019 alone. Despite this burden, community-level mitigation strategies remain underdeveloped. Community health volunteers (CHVs), embedded within their communities and trusted by populations with limited healthcare access, represent an underutilised but promising platform for AMR mitigation. This scoping review aimed to map the breadth, nature, and outcomes of CHV-led or CHV-supported AMR interventions in SSA; identify barriers and facilitators to implementation; and identify evidence gaps to inform future research and policy. Following the Arksey and O’Malley (2005) framework and reported according to PRISMA-ScR 2018 guidelines, we systematically searched PubMed/MEDLINE, EMBASE, Cochrane Library, AJOL, WHO IRIS, and grey literature for studies published from January 2018 to January 2026. Studies conducted in SSA involving CHVs and addressing AMR mitigation through at least one of education, surveillance, diagnostics, waste management, or One Health approaches were eligible. Data were charted using a standardised extraction form and findings synthesised narratively. Of 847 records identified, 26 studies and reports met the inclusion criteria. The evidence base encompassed educational interventions (n = 14), diagnostic integration (n = 3), waste and environmental management (n = 3), and multisectoral One Health approaches (n = 6). CHV-led educational interventions improved AMR knowledge by 49.3%–97.1% and reduced inappropriate antibiotic prescribing by 18–44%. Point-of-care diagnostic integration reduced antibiotic use by up to 24.6% without increasing adverse outcomes. Key barriers included inadequate resources, training overload, weak regulation, and lack of integrated surveillance, while facilitators included tailored training, diagnostic tools, community trust, and policy alignment. The evidence supports embedding structured AMR roles for CHVs within national AMR action plans, integrated community case management, One Health frameworks, and investment in CHV training and diagnostic capacity.

## Introduction

### The AMR crisis in Sub-Saharan Africa

Antimicrobial resistance (AMR) has emerged as one of the most formidable threats to global public health in the twenty-first century. In 2019 alone, bacterial AMR was directly responsible for an estimated 1.27 million deaths worldwide and associated with approximately 4.95 million deaths [1]. These figures surpass the mortality burden of HIV/AIDS or malaria, redefining AMR as a leading cause of death globally. Crucially, the burden is not equitably distributed: all four sub-regions of SSA had all-age death rates associated with bacterial AMR exceeding 75 per 100,000 population, with western SSA bearing the highest burden globally at 27.3 attributable deaths per 100,000 [[1] Antimicrobial Resistance Collaborators, 2022]. The WHO African Region alone documented approximately 255,000 direct AMR deaths in 2019 [2]. Forecasting models project that without decisive intervention, cumulative AMR-attributable deaths in SSA could rise to 6.63 million between 2025 and 2050 [3].

The drivers of AMR in SSA are deeply entrenched and multifactorial. Widespread misuse of over-the-counter availability of antibiotics without prescription, inadequate water, poor sanitation, and hygiene (WASH) infrastructure, unsupervised antibiotic use in livestock and agriculture, weak regulatory frameworks, and fragile health systems collectively create an environment highly conducive to resistance selection and transmission [4,5]. In SSA’s abundant peri-urban and rural settings, informal medicine sellers, traditional healers, and community-level drug retailers are often the first (and sometimes sole) point of contact for sick individuals, bypassing trained healthcare professionals entirely [6,7]. Within these spaces, community-level acquisition drives AMR spread: invasive AMR infections in SSA frequently originate not from hospitals but from community reservoirs, as demonstrated by high community carriage rates of extended-spectrum beta-lactamase-producing *Enterobacterales* (ESBL-E) in countries including Burkina Faso, Malawi, and Uganda [8,9].

Despite this epidemiological reality, AMR mitigation strategies in SSA are below expectations, and if present, they have remained overwhelmingly hospital- and facility-centric. The landmark scoping review by Kamere et al. [10] examining national AMS activities across eight African countries confirmed well-established national AMR coordination committees and action plans, but revealed starkly inconsistent implementation, critical surveillance gaps, and a near-complete absence of formalised community-level stewardship structures. This facility bias leaves the vast majority of SSA populations who live, acquire infections, and access treatment outside formal health facilities, essentially unaddressed by existing AMR containment efforts.

### Community health volunteers in SSA

Community health volunteers (CHVs), variously termed community health workers (CHWs), village health workers, or lay health promoters, form the backbone of primary healthcare delivery in much of SSA. **Definitions of CHVs vary across SSA, but they are broadly understood as community members with some level of health training who serve as a bridge between communities and formal health systems, with roles, remuneration, and integration into health systems differing substantially across contexts** [11,12]. Typically recruited from within and trained to serve their own communities, they bridge the persistent gap between peripheral health facilities and the populations they serve. They are trusted intermediaries, culturally embedded, conversant in local languages and norms, and capable of reaching populations that formal health systems do not. In Kenya alone, over 100,000 trained CHVs are integrated into the government health system [13]. Across SSA, CHVs already perform critical functions including health education, disease surveillance, case identification, treatment support, and referral, all directly relevant to AMR mitigation.

The WHO has explicitly recognised the potential of CHWs in AMR mitigation, publishing a technical brief in 2021 advocating inclusion of AMR and antimicrobial stewardship (AMS) competencies in CHW training curricula [14]. UNICEF’s guidance on AMR and child health highlights integrated community case management (iCCM) as a critical platform for rational antibiotic use in children in LMICs, noting that CHWs managing pneumonia in Zambia achieved 92% guideline adherence [15]. The One Health Joint Plan of Action 2022–2026, developed by the Quadripartite (WHO, FAO, WOAH, UNEP), further positions community engagement as a core pillar of the global AMR response [16].

Despite these policy signals, the evidence base underpinning CHV-led AMR interventions in SSA remains poorly characterised. Existing reviews have been narrowly focused, addressing specific countries, single intervention types, or general healthcare worker training, and fail to comprehensively map CHV-specific AMR activities [17,10]. No prior review has examined CHV-led interventions through an explicit One Health lens encompassing human, animal, and environmental health domains simultaneously, which are documented as key pillars in AMR dynamics [18,19]. The systematic review by Tumwine et al. [20] marks the closest predecessor, yet gaps remain in synthesising quantitative outcomes, digital and diagnostic dimensions, the COVID-19 pandemic context, and grey literature contributions that together define the current evidence landscape.

### Rationale and objectives

This scoping review was conducted to address these gaps. It recognises that a scoping review is particularly suited to mapping broad, heterogeneous evidence fields and identifying research lacunae, as opposed to answering narrow effectiveness questions. We adopted the Arksey and O’Malley [21] framework, enhanced by Levac et al. [22]. Our approach captures not only the breadth but also the depth of CHV-AMR activities across SSA, includes both published and grey literature, and applies an explicit One Health framework throughout.

The primary objectives of this scoping review were to: (1) map the scope, types, settings, and outcomes of CHV-led or CHV-supported AMR interventions across SSA; (2) identify barriers and facilitators to CHV engagement in AMR mitigation; (3) assess the degree to which interventions incorporate One Health principles; and (4) delineate evidence gaps to guide future research priorities and policy development.

Our guiding research questions were: (a) What interventions have CHVs been involved in to address AMR in SSA, and what reported outcomes? (b) What barriers and facilitators influence CHV engagement in AMR mitigation? (c) To what extent do existing CHV-led AMR interventions integrate One Health principles? (d) What are the critical gaps in evidence that impede the scaling of CHV-led AMR programmes in SSA?

## Methods

This scoping review was conducted in accordance with the Arksey and O’Malley [21] framework, as refined by Levac et al. [22], and is reported in conformance with the Preferred Reporting Items for Systematic Reviews and Meta-Analyses extension for Scoping Reviews (PRISMA-ScR) [23] (S1 Checklist). The review was retrospectively registered on the Open Science Framework prior to resubmission (https://doi.org/10.17605/OSF.IO/26KHP).

### Eligibility criteria

Inclusion and exclusion criteria were defined a priori using a Population-Concept-Context (PCC) framework.

*Population.* Studies involving CHVs, CHWs, lay health workers, village health workers, community animal health workers (CAHWs), or equivalent community-level cadres operating in SSA were eligible. Studies where CHVs played a peripheral or entirely absent role were excluded, as were studies exclusively involving facility-based workers without a community-level component and editorials, commentaries, or conference abstracts without extractable data.

*Concept.* The primary concept was CHV involvement in AMR mitigation, broadly defined to include: (i) antibiotic stewardship activities; (ii) infection prevention and control (IPC) promotion; (iii) surveillance or case identification; (iv) diagnostic tool integration; (v) antimicrobial waste management; and (vi) One Health activities bridging human, animal, and environmental health domains.

*Context.* All 47 countries in SSA were eligible, with no restrictions on setting (urban, peri-urban, or rural) or publication status. Searches were limited to January 2018 to January 2026. This start date was chosen because global recognition of CHVs in AMR interventions gained traction only after the 2016 UNGA High-Level Meeting on AMR, which triggered a series of milestones including national AMR action plans, the WHO Global Action Plan rollout (2017–2019), and the formal integration of CHWs into AMR discourse in the UNGA Political Declaration (2024) [24] and a WHO Technical Brief (2025) [25,26]. Pre-2018 evidence was therefore considered unlikely to reflect current programme designs or policy contexts; this restriction is acknowledged as a limitation.

### Information sources and search strategy

Searches were conducted in January 2026 across five electronic databases: PubMed/MEDLINE, EMBASE (via Ovid), Cochrane Library (including the Cochrane Database of Systematic Reviews and CENTRAL), African Journals Online (AJOL), and WHO IRIS. These were supplemented by grey literature sources comprising ReAct Africa [27,28], the AMREF document repository, UNICEF technical guidance, WHO and Quadripartite AMR publications, and national AMR action plans from SSA countries.

The search strategy was developed in consultation with an information specialist and comprised MeSH terms and free-text terms combined with Boolean operators. Key search concepts covered three domains: CHV cadre terminology (including CHV, CHW, lay health worker, village health worker, health extension worker, and community animal health worker); AMR-related terminology (including antimicrobial resistance, antibiotic stewardship, antimicrobial stewardship, antibiotic use, infection prevention and control, and rational use); and geographic scope (all 47 SSA countries by name alongside the MeSH term Africa South of the Sahara). The search strategy for PubMed is provided in S1 Text.

#### Study selection.

A total of 847 records were identified through database and grey literature searches. All records were imported into Rayyan for deduplication and two-stage screening. After removal of 214 duplicates, 633 records underwent title and abstract screening by two independent reviewers. Of these, 541 were excluded, leaving 92 records for full-text assessment. Disagreements during screening were resolved through discussion, with unresolved conflicts adjudicated by a third reviewer.

**Fig 1:**
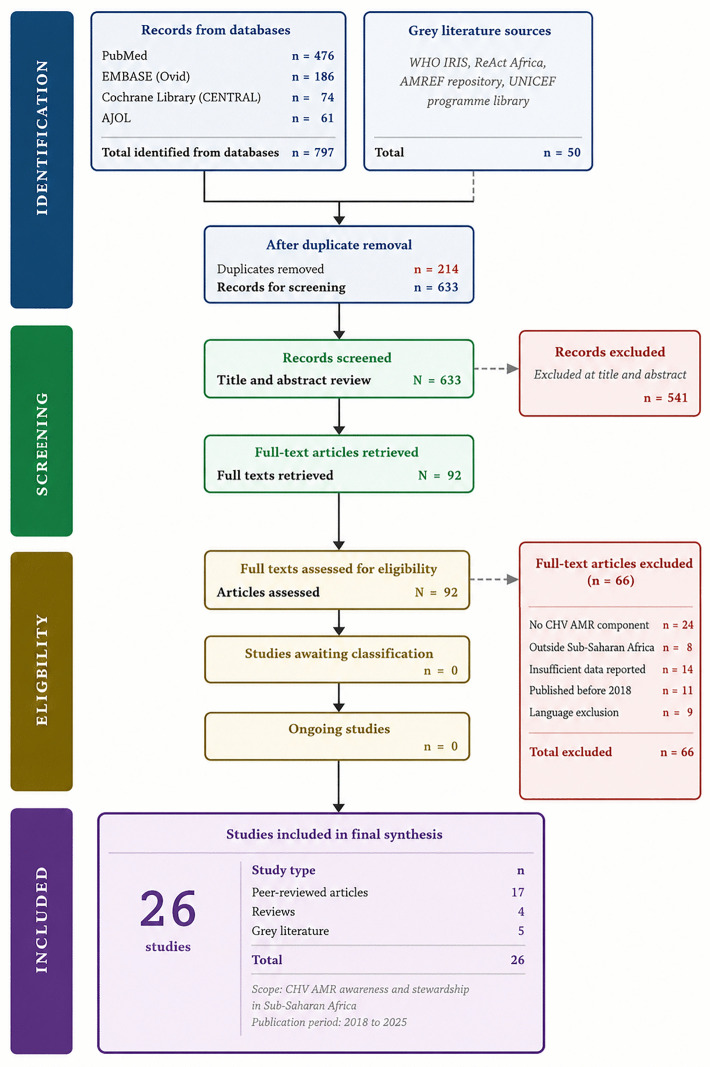
PRISMA-ScR flow diagram of study selection.

Following full-text review, 66 records were excluded for various reasons. These included a lack of a CHV–AMR component, insufficient data, or being conducted outside Sub-Saharan Africa. Twenty-six sources met the inclusion criteria and were included in the final synthesis. These comprised 17 peer-reviewed empirical studies, 4 systematic/scoping reviews, and 5 grey literature or policy documents. Fig 1 below is a PRISMA Flow chart summarizing the process.

#### Data charting.

Data were extracted using a standardised charting form piloted on five records. Extracted fields included: study citation and DOI; year; country and setting; study design; population and sample characteristics; CHV cadre and role; intervention type and components; AMR mitigation domain(s); One Health elements; outcome measures; key findings; and reported barriers and facilitators. Extraction was performed independently by two reviewers with discrepancies resolved by consensus. Consistent with scoping review methodology, formal quality appraisal of included studies was not undertaken; methodological characteristics were charted descriptively [29].

### Synthesis

Given the heterogeneity of included evidence, findings were synthesised narratively. Evidence was categorised by intervention type (educational, diagnostic, waste management, and One Health multisectoral) and mapped against AMR mitigation domains. Barriers and facilitators were synthesised thematically using a framework adapted from the Consolidated Framework for Implementation Research [CFIR; 30]. One Health integration was assessed using a three-tier taxonomy: human health only; human and animal health; and full One Health (human, animal, and environmental).

## Results

### Characteristics of included studies

The 26 included sources spanned 12 SSA countries: Uganda (n = 7), Kenya (n = 4), Ghana (n = 3), Tanzania (n = 2), Malawi (n = 3), Zambia (n = 2), Ethiopia (n = 2), Sierra Leone (n = 1), Zimbabwe (n = 1), Burkina Faso (n = 1), and multi-country studies (n = 2); grey literature documents covered SSA broadly. Studies were published between 2018 and 2025. Study designs included randomised/stepped-wedge trials (n = 3), quasi-experimental designs (n = 4), cross-sectional mixed-methods (n = 5), qualitative studies (n = 7), narrative and scoping reviews (n = 4), and grey literature/policy documents (n = 5). Table 1 provides a detailed summary of included sources.

**Table 1:** Characteristics of Included Studies (n = 26).

Reference	Country/Setting	Design	Population	Intervention Type	One Health Level	Key Findings
Ciccone et al. [31]	Uganda (Rural)	Stepped-wedge RCT	Children/CHWs	Diagnostic (CRP POC)	One Health element is absent	24.6% reduction in antibiotic use; no adverse outcomes
Musoke et al. [32]	Uganda (Wakiso)	Mixed-methods	227 CHWs	Educational/One Health	Full One Health	Knowledge: 49.3% → 97.1%; 81% improved handwashing
Musoke et al. [33]	Uganda (Wakiso)	Qualitative cross-sectional	CHWs/community	Waste management	Partial One Health	CHWs used safety boxes; travel barriers identified
Nyamu et al. [34]	Kenya (Rural)	Before-after quantitative	3,014 patients	Educational (training)	Human health only	Antibiotic prescriptions ↓44% (children <5); ↓ 18% older children
Graham et al. [35]*	Zambia	Cross-sectional mixed-methods	90 CHWs	Educational (iCCM)	Human health only	92% guideline adherence; 65% confirmed pneumonia Rx
Virhia et al. [36]	Tanzania	Qualitative	44 participants	Educational	Human health only	Variable AMR understanding; structural challenges
Abuya et al. [37]	Kenya (Urban/Rural)	Cross-sectional	CHWs	Case identification/scaling	Human health only	Improved guideline scaling for PSBI
Tiruneh et al. [38]	Ethiopia (Rural)	Quasi-experimental	CHWs	PSBI treatment/pandemic	Human health only	Increased PSBI treatment coverage during COVID-19
Mankhomwa et al. [39]	Malawi (Urban)	Qualitative cross-sectional	CAHWs	Surveillance/dispensing	Human+Animal	CAHWs dispensed antibiotics; practices fuel AMR
Kaawa-Mafigiri et al. [40]	Uganda	Mixed-methods qualitative	HWs/outpatients	Training/communication	Human health only	Improved adherence; reduced sharing/incomplete doses
Appiah et al. [41]	Ghana	Mixed-methods	School children	Educational (community)	Human health only	Picture drawing: positive knowledge/attitude impact
Tsekleves et al. [42]	Ghana	Mixed-methods	Households	Community engagement/hygiene	Human+Environment	New hygiene strategies adopted post-workshop
Hamilton & Bugg [43]	Sierra Leone	Cross-sectional	Community health officers	Educational/guideline	Human health only	85% appropriate prescribing at 1 week; ↓ 65% at 2 months
Dixon et al. [6]	Malawi/Uganda/Zimbabwe	Mixed-methods anthropological	Households	Surveillance/informal provision	Human health only	Informal sources of antibiotics due to stockouts
Godman et al. [4]	SSA (multi-country)	Narrative review	Stakeholders	Policy/ASPs/CHW roles	One Health implied	Variable NAP implementation; CHW roles indirect
Kamere et al. [10]	8 African countries	Scoping review	National stakeholders	AMS landscape/One Health	One Health framework	All countries have AMRCCs; implementation gaps persist
Sartorius et al. [2]	WHO African Region	Systematic analysis	Population-level	AMR burden estimation	N/A (epidemiological)	High AMR burden in Africa; SSA is most affected
Tumwine et al. [20]	Africa	Systematic review	CHWs/CAHWs	AMS roles in Africa	Full One Health	CHVs are key to community AMS; evidence gaps identified
WHO [14]	Global/SSA examples	Technical brief	CHWs/policymakers	Training/curricula	One Health implied	Essential to embed AMR in CHW curricula
UNICEF [15]	Global/SSA focus	Guidance note	Children/CHWs/iCCM	iCCM/rational use	Human health focus	Children bear ~20% AMR deaths; iCCM key platform
ReAct Africa [44] [27,28]	SSA (Kenya focus)	Programme report	Youth/CHWs	Advocacy/campaigns	Human health	Youth/CHW campaigns scale AMR action
Kenya MoH [13]	Kenya	NAP document	All sectors	Policy framework	Full One Health	CHVs are involved in the awareness/education pillars
AMREF [45]	SSA (Kenya/Ethiopia)	Programme report	CHWs/communities	AMR landscape/CHW training	One Health implied	CHW training in health systems; AMR projects
Quadripartite [46]	Global/SSA progress	Biennial report	All sectors	GAP-AMR monitoring	Full One Health	Community education implied; funding gaps remain
Valia et al. [7]	Burkina Faso (Rural)	Cross-sectional	Hospital patients/community	Antibiotic use patterns	Human health	High OTC antibiotic use in the community; the CHW role is implied
James et al. [47]	Global/SSA context	Narrative review	All sectors	One Health AMS strategies	Full One Health	Sociocultural and structural barriers key in LMICs

**Note.** CHWs = community health workers; CAHWs = community animal health workers; RCT = randomised controlled trial; iCCM = integrated community case management; PSBI = possible serious bacterial infection; CRP = C-reactive protein; POC = point-of-care; AMS = antimicrobial stewardship; AMR = antimicrobial resistance; NAP = national action plan; AMRCC = AMR coordinating committee; * Graham et al. [35] *included due to direct relevance despite pre-2018 date.*

### Intervention types and key findings

#### Educational and training interventions.

The most frequently represented intervention type was CHV-focused education and training (n = 14 sources). The most compelling evidence came from Musoke et al. [32], who implemented a One Health-informed workshop series for 227 CHWs in Wakiso District, Uganda, using the COM-B behaviour change model. Pre-post assessments revealed that knowledge of AMR definition improved from 49.3% to 97.1%, and 90.3% of CHWs reported improved AMS practices post-intervention, including an 81% increase in hand washing. Also, Nyamu et al. [34] reported that interactive AMS and guideline training in rural Kenya reduced antimicrobial prescriptions for respiratory tract infections by 44% in children under five years and 18% in older children within two weeks, with 3,014 patient records analysed. Graham et al. [35], on the other hand, found that six-day iCCM training in Zambia enabled 90 CHWs to achieve 92% adherence to antibiotic prescribing guidelines for paediatric pneumonia. Hamilton and Bugg [43] reported that a prescribing guideline for community health officers in Sierra Leone achieved 85% appropriate prescribing at one week, declining to 65% after two months, underscoring that one-time training without follow-up supervision is insufficient for sustained behaviour change.

Kaawa-Mafigiri et al. [40] evaluated a tailored training and communication package for health workers and outpatients in Uganda using pictorial messaging, finding significant improvements in antibiotic adherence and a reduction in antibiotic sharing and incomplete dosing. Virhia et al. [36], in Tanzania, found considerable variation in AMR knowledge translation and identified structural barriers. These barriers included patient-blaming narratives. Altogether, these constrained effective community education, reinforcing calls for context-sensitive, non-stigmatising training approaches. Appiah et al. [41] innovatively assessed storytelling and picture drawing as AMR awareness tools among children in Ghana, finding that picture drawing positively impacted AMR knowledge and attitudes, whereas storytelling had a statistically significant negative effect on knowledge.

#### Diagnostic integration.

Ciccone et al. [31] conducted a stepped-wedge cluster randomised trial in rural Uganda assessing whether CHW-administered point-of-care C-reactive protein (CRP) testing could safely reduce antibiotic use among children with respiratory illness. The CRP-guided algorithm significantly reduced antibiotic use by 24.6% without increasing adverse patient outcomes, with high CHW adherence to the clinical guidance protocol. This landmark trial represents the strongest evidence to date for the role of CHV-delivered diagnostics in AMR mitigation in SSA, demonstrating that CHVs can safely and effectively employ biomarker-guided decision-making to rationalise antibiotic prescribing even in resource-constrained rural settings. Abuya et al. [37] and Tiruneh et al. [38] further demonstrated CHW roles in PSBI case identification and maintenance of treatment coverage during the COVID-19 pandemic.

#### Antimicrobial waste and environmental management.

Musoke et al. [33] qualitatively explored antimicrobial access, use, and disposal in Wakiso District, Uganda, finding that CHWs used safety boxes for medical waste management, though persistent barriers, including long travel distances to disposal sites, and limited effectiveness. Tsekleves et al. [42] engaged households in Ghana through a community co-design process to develop home cleaning interventions targeting bacterial contamination, finding that a 30-day cleaning contract with community workshops prompted positive hygiene behaviour changes. In one study done in Malawi, Mankhomwa et al. [39] found that the community animal health workers (CAHWs) dispensed antibiotics without standardised guidance in ways that contributed to AMR drivers, but also identified CAHWs as critical nodes for One Health stewardship reform in the agricultural sector.

#### One Health multisectoral interventions.

Six sources explicitly adopted or discussed One Health frameworks spanning human-animal-environment interfaces. The Musoke et al. [32] study represents the most operationalised One Health CHV-AMR training programme documented in the peer-reviewed literature. The Kamere et al. [10] review confirmed the widespread adoption of One Health AMR coordination committees across eight African countries, but documented that human health sector activities dominate implementation, whilst animal and environmental sector integration remains limited. Godman et al. [4] identified CHW vaccination campaigns as an indirect One Health AMR mitigation strategy. They work by reducing infection incidence, antibiotic demand is correspondingly reduced. The Quadripartite biennial report [46] and James et al. [47] both emphasise that CHV programmes are uniquely positioned to bridge policy and practice in One Health AMR strategies (Table 2).

**Table 2:** Synthesis of AMR mitigation outcomes by intervention domain.

Intervention Domain	Countries/Settings	Study Designs	Key Quantitative/Qualitative Outcomes	Interpretation
Educational/Training	Uganda, Kenya, Zambia, Sierra Leone, Ghana, Tanzania	Pre-post; cross-sectional; qualitative	AMR knowledge: 49.3% → 97.1% [32]; antibiotic prescriptions ↓18–44% [34]; guideline adherence 85–92% [43,35]	Strong short-term knowledge/behaviour gains; sustainability requires structured supervision and repeated training cycles
Diagnostic Integration	Uganda, Kenya, Ethiopia	RCT; quasi-experimental; cross-sectional	CRP-guided algorithm ↓antibiotic use 24.6% safely [31]; improved PSBI coverage [37,38]	Highest-quality evidence; CHVs can safely execute POC diagnostics; pandemic resilience demonstrated
Waste/Environmental Management	Uganda, Ghana, Malawi	Qualitative; community co-design	Safety box use for waste [33]; hygiene behaviour changes post-workshop [42]; CAHW dispensing practices documented [39]	Environmental AMR mitigation is achievable via CHVs; the critical CAHW role in agricultural AMR; barriers include infrastructure and distance.
One Health Multisectoral	Uganda, Multi-country SSA	Mixed-methods; scoping review; policy documents	COM-B behaviour change gains across human+animal+environment domains [32]; CHVs identified as key actors in 5-country landscape [45]; policy frameworks endorse CHV One Health roles (Kenya NAP, 2023)	One Health integration at the community level is feasible; policy frameworks are supportive; implementation remains nascent and underfunded.
Policy/Systemic Level	SSA (multiple)	Narrative review; systematic analysis; programme reports	All 8 surveyed countries have AMRCCs; variable NAP implementation [10]; iCCM platforms endorsed for CHV-AMR integration [15,14]	Policy infrastructure exists, but community operationalisation lags; grey literature is critical for identifying programmatic realities.

### Synthesis of outcomes across intervention types

**Fig 2:**
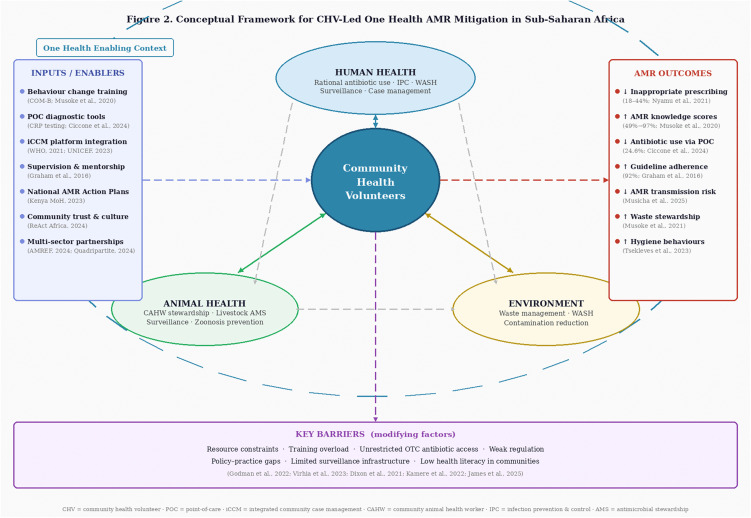
Conceptual framework for CHV-led one health AMR mitigation in Sub-Saharan Africa. **Note.** *CHV = community health volunteer; POC = point-of-care; iCCM = integrated community case management; CAHW = community animal health worker; IPC = infection prevention and control; AMS = antimicrobial stewardship; WASH = water, sanitation, and hygiene. Bidirectional solid arrows denote CHV interaction with each One Health domain. Dashed grey arrows indicate inter-sectoral pathways. Dashed coloured arrows represent flow from enablers to CHVs (left panel) and from CHVs to outcomes (right panel). Barrier modifiers (bottom panel) exert a negative influence throughout the system*.

### Barriers and facilitators

A thematic analysis of barriers and facilitators to CHV engagement in AMR mitigation across included sources identified five barrier themes and four facilitator themes, summarised in Table 3.

**Table 3:** Barriers and facilitators to CHV-led AMR interventions in SSA.

Theme	Description	Key Supporting Evidence
**BARRIERS**		
**Resource Constraints**	Inadequate financing, limited diagnostic supplies, and infrastructure deficits	Musoke et al. [33]: travel distances to waste disposal; Godman et al. [4]: funding gaps in Zimbabwe (>US$7.5M/year needed) and Ghana ($21M for 5-year NAP); AMREF [45]: persistent resource constraints across 5 countries
**Training Overload**	CHVs already bear a high task burden; AMR added without reducing other duties.	WHO [14]: risk of overburdening CHWs with curricula additions; Virhia et al. [36]: structural barriers limiting knowledge translation in Tanzania
**Regulatory and Supply Gaps**	Unrestricted OTC antibiotic access; informal medicine sellers bypassing prescribers	Dixon et al. [6]: reliance on informal sources due to facility stockouts; Valia et al. [7]: high OTC antibiotic use in the Burkina Faso community
**Knowledge and Attitude Gaps**	Variable AMR understanding; patient-blaming narratives; low health literacy	Virhia et al. [36]: variation in AMR knowledge translation in Tanzania; Kaawa-Mafigiri et al. [40]: comprehension barriers among community members
**Policy-Practice Gaps**	National frameworks are not operationalised at the community level; CHV AMR roles are not formalised.	Kamere et al. [10]: all 8 countries have AMRCCs but community implementation lags; Kenya NAP (2023): CHV roles implied but not explicitly mandated
**FACILITATORS**		
**Tailored CHV Training**	Context-sensitive, culturally appropriate, multi-modal training using behaviour change frameworks	Musoke et al. [32]: COM-B model training achieving >97% post-training knowledge; Nyamu et al. [34]: interactive guideline training ↓prescriptions 44%
**Diagnostic Tool Integration**	Point-of-care testing empowering CHVs with decision-support tools	Ciccone et al. [31]: CRP POC testing ↓antibiotic use 24.6% safely; Abuya et al. [37]: case identification tools improving guideline scaling
**Community Trust and Social Capital**	CHV embeddedness in communities enabling health education uptake	Graham et al. [35]: 92% guideline adherence by trained CHWs; ReAct Africa [27]: youth/CHW community ambassador model scaling AMR action in Kenya
**Policy and Programmatic Alignment**	Integration of CHV AMR roles into existing national frameworks (iCCM, NAPs, One Health)	UNICEF [15]: iCCM platforms as natural AMR integration vehicles; Kenya NAP (2023): One Health partnerships include AMREF/ILRI supporting CHV contexts; WHO [14]: technical brief advocating CHW AMR curricula inclusion

### One Health integration: A three-tier assessment

We assessed the degree of One Health integration across all 26 included sources using a three-tier taxonomy. Only five sources demonstrated full One Health integration: Musoke et al. [32], Kamere et al. [10], Tumwine et al. [20], Kenya NAP (2023), and the Quadripartite biennial report [46]. Eight sources achieved partial One Health integration. The remaining 13 sources focused exclusively on human health, confirming that full One Health integration at the community CHV level remains an aspirational rather than operationalised goal across most of SSA.

This finding has important implications. The persistence of multidrug-resistant pathogens in community settings is inseparable from livestock management, antibiotic use in agriculture, and environmental contamination routes. ESBL-E transmission across human-animal-environment compartments in eastern Africa has been directly documented via whole-genome sequencing [8]. The cluster-RCT by Ingelbeen et al. [48] demonstrated that an integrated WASH and antibiotic behaviour change community intervention produced modest but consistent reductions in ESBL-E household acquisition (hazard ratio 0.82 at months 6–9), underscoring the multifactorial nature of community AMR transmission and the need for integrated CHV programme designs.

### Evidence gaps identified

Across all 26 included sources, several critical evidence gaps were consistently identified. First, there is an absence of rigorously designed trials measuring CHV-led AMR intervention impact on microbiologically confirmed resistance outcomes. Second, no study has conducted a formal cost-effectiveness analysis of CHV-led AMR programmes in SSA. Third, the role of digital health tools in enhancing CHV AMR capacity was referenced programmatically but not empirically evaluated in any included study. Fourth, long-term follow-up data on the durability of behaviour change following CHV training are largely absent. Fifth, francophone SSA countries are disproportionately underrepresented in the evidence base.

## Discussion

### Principal findings

This scoping review maps CHV-led AMR interventions across SSA within a One Health framework, drawing on 26 sources from 12 countries. CHV-delivered education and training reliably improved AMR knowledge and antibiotic prescribing behaviour. Point-of-care diagnostic integration provided the most rigorous evidence for direct antibiotic use reduction. However, these gains were documented under specific programme conditions and should not be uncritically generalised across SSA’s diverse health system landscapes, including from Uganda to other contexts. Effectiveness is shaped by multiple macro and micro level determinants, and contextually tailored training remains inconsistently implemented across the region. One Health approaches, though present in only a minority of programmes, demonstrated synergistic gains across human, animal, and environmental domains. CHVs are nonetheless generally less equipped to address agricultural and environmental AMR dimensions without substantially more integrated training, cross-sectoral collaboration, and dedicated resourcing. The community-level gap in SSA’s AMR response is both well-evidenced and actionable.

The WHO has advocated for rapid, accurate diagnostic tools as a key strategy to reduce unnecessary antibiotic prescribing, emphasising that timely, biomarker-guided decision-making can substantially curb AMR [14,31]. The findings synthesised in this review corroborate this position: CHV-administered point-of-care diagnostics not only improved clinical management of common illnesses but demonstrably reduced inappropriate antibiotic use [31]. Expanding access to simple, accurate, low-cost diagnostic tools that are designed for use by community-level cadres, therefore, represents a high-priority investment for AMR mitigation in SSA [49].

Improper disposal of antimicrobials and antimicrobial-contaminated waste is a well-documented contributor to environmental AMR reservoirs, facilitating horizontal gene transfer and persistence of resistant strains in soil and water [33]. The evidence synthesised in this review indicates that structured CHV engagement in antimicrobial waste management can meaningfully reduce these environmental AMR pathways, when supported by clear protocols and adequate infrastructure. However, this potential is contingent on sustained training, functional waste disposal systems, and regular supervision. Community animal health workers (CAHWs) represent an underutilised but critical cadre for extending stewardship into the agricultural sector; targeted training and government-supported integration of CAHWs into One Health AMR programmes is a priority [20].

Our review directly addresses a gap in the literature that previous reviews have not filled. The Tumwine et al. [20] systematic review provides an important foundation on CHW/CAHW AMS roles in Africa, but our scoping approach permits a broader conceptualisation of AMR mitigation that goes beyond AMS narrowly defined to encompass surveillance, waste management, diagnostic integration, and community engagement. The Kamere et al. (2022) scoping review of national AMS activities maps the policy landscape but does not focus on community implementation. Our review uniquely synthesises these dimensions and extends to grey literature.

### Interpretation in light of existing literature

The global AMR burden data from the Antimicrobial Resistance Collaborators [1] and the Africa-specific analysis of Sartorius et al. [44] frame the urgency of our findings with unambiguous epidemiological force. With 255,000 direct AMR deaths in the WHO African Region in 2019, and cumulative SSA projections approaching 6.63 million deaths by 2050 [3], the current under-investment in community-level AMR mitigation strategies represents not merely a programmatic gap but a preventable mortality burden of significant and growing magnitude. The evidence from this review suggests that CHVs, appropriately trained and equipped, can substantively reduce this burden through multiple complementary pathways.

At the same time, the broader literature cautions against overstatement. CHW interventions are clearly beneficial at the community level, but their impact is constrained by structural and systemic factors that are not amenable to CHV-led action alone. Power differences, poverty, and deep-rooted vulnerabilities may be systemically embedded in ways that limit what community-level actors can achieve without upstream structural reform [50]. The heterogeneity of CHV roles and cadres across SSA further complicates generalisation: who CHVs are, what they are trained to do, how they are remunerated, and how they are integrated into health systems varies substantially across and within countries [11]. This heterogeneity must be explicitly acknowledged in any attempt to translate findings into policy.

The consistency of educational intervention outcomes across diverse SSA settings suggests that CHV-led AMR education, when grounded in behaviour change theory and adapted to local context, generates reproducible and meaningful impacts. The COM-B model applied by Musoke et al. [[51,50]] offers a particularly promising theoretical architecture for future CHV AMR training, as it explicitly addresses capability, opportunity, and motivational determinants of behaviour rather than treating knowledge transfer as an end in itself. However, even well-designed behaviour change models have limitations, and the COM-B framework is no exception. Structural barriers such as antibiotic availability without prescription, stockout-driven reliance on informal suppliers, and insufficient supervision can undermine behaviour change gains that appear robust in short-term post-training assessments. AMR is itself a wicked problem, characterised by ethical tensions, political complexity, and cross-sectoral interdependencies that resist straightforward technical solutions [50,52]. CHV programmes must therefore be positioned as one necessary component of a broader systemic response, not as a standalone solution. This aligns with the broader implementation science literature, which consistently demonstrates that knowledge alone is insufficient to drive sustained behaviour change in complex social environments [47].

The Ciccone et al. [31] stepped-wedge RCT from Uganda represents the strongest evidence, showing that point-of-care diagnostic empowerment of CHVs has significant practical implications. It enables CHVs to move beyond health education toward evidence-guided clinical decision support, with antibiotic prescribing informed by biomarker results rather than clinical impression alone. This aligns with the WHO AWaRe framework, which prioritises appropriate use of Access antibiotics and restriction of Watch and Reserve categories. These are goals that community-level diagnostics can meaningfully support, where infrastructure permits.

The evidence on One Health integration is both promising and sobering. The documented transmission of ESBL-producing E. coli across human-animal-environment compartments in SSA [8] makes a compelling epidemiological argument that CHV programmes addressing only human antibiotic use will leave significant AMR drivers unaddressed. The Musoke et al. [32] One Health CHW programme in Uganda was the only study with robust empirical data on full One Health CHV AMR training. It demonstrated that CHVs can successfully engage with cross-sector content, including animal health and environmental hygiene. Yet this single study cannot bear the weight of policy prescriptions for the entire region. The global AMR burden has been described as a super-wicked problem requiring intersectoral collaboration, stakeholder alignment, and long-term systemic transformation [50]. Community-level interventions, however well designed, must contend with the ethical, political, and economic forces that drive antimicrobial misuse across the human-animal-environment interface.

### Implications for practice and policy

For health practitioners and programme managers, our findings translate into several concrete recommendations. First, AMR and AMS modules should be systematically incorporated into CHV pre-service and in-service training curricula across SSA, as advocated by the WHO [53,14] technical brief but as yet inconsistently implemented. Secondly, diagnostic empowerment of CHVs through access to point-of-care tests should be prioritised in settings where respiratory infections and febrile illness drive high antibiotic demand, as demonstrated by the Ciccone et al. [31] trial. Thirdly, existing iCCM platforms represent the most natural and cost-efficient vehicle for integrating CHV-led AMR stewardship. Fourth, community-level One Health approaches that explicitly involve both human health CHVs and community animal health workers (CAHWs) in shared AMR messaging and surveillance activities should be piloted and evaluated. Any such pilots must be accompanied by rigorous evaluation frameworks, given the current absence of microbiologically confirmed endpoint data from CHV-led AMR programmes in SSA.

For policymakers, our review highlights the critical gap between the strong policy rhetoric supporting community engagement in national AMR action plans and the limited evidence of operationalisation at the CHV level. The Kenya NAP on AMR 2023–2027 exemplifies this gap: while it acknowledges community education and awareness as strategic pillars, explicit role definitions and resourcing for CHVs remain absent. Translating the Quadripartite One Health Joint Plan of Action’s community engagement ambitions into funded, evaluated CHV programmes requires political will, multi-sectoral coordination, and dedicated community health system financing. The 2024 United Nations Political Declaration on AMR, which commits member states to reducing AMR-attributable mortality by 10% by 2030, provides an important policy anchor for this work [24]. However, meeting these targets in SSA, where the burden is highest and health system capacity most constrained, will require translating such declarations into concrete, funded, community-level action.

### A proposed conceptual framework for CHV-led One Health AMR mitigation

Drawing on the synthesis of evidence presented in this review, we propose a conceptual framework for the operationalisation of CHV-led AMR mitigation in SSA within a One Health context (Fig 2). The framework is grounded in three established theoretical foundations: (i) the COM-B behaviour change model [54], as applied empirically by Musoke et al. [32] in Uganda, which positions CHV capability, opportunity, and motivation as the proximal determinants of effective AMR stewardship behaviour; (ii) the One Health Joint Plan of Action’s community engagement pillar [16], which situates CHVs at the nexus of human, animal, and environmental health domains; and (iii) the Consolidated Framework for Implementation Research [CFIR; 30], which provides the structural logic for understanding how outer context (policy, partnerships, national AMR plans) and inner context (community trust, CHV capacity, local health system integration) jointly determine implementation success.

It is important to acknowledge that this framework, like the COM-B model on which it partly rests, remains theoretical. It is grounded in the best available evidence, but the evidence base is limited by small study samples, short follow-up periods, heterogeneous outcome measures, and near-total absence of microbiological endpoints. The framework should therefore be treated as a hypothesis-generating tool and a guide for programme design, not as a proven blueprint. As depicted in Fig 2, CHVs occupy the central hub of the framework, positioned as bidirectional actors within all three One Health domains simultaneously. In the human health domain, CHVs deliver AMS education, support rational antibiotic use, promote IPC and WASH practices, and facilitate case identification and referral. These functions are already embedded in iCCM platforms endorsed by both WHO [14] and UNICEF [15]. In the animal health domain, community animal health workers (CAHWs) represent the equivalent cadre and have been documented both as contributors to AMR through unregulated dispensing [39] and as critical nodes for One Health stewardship reform [20,55]. In the environmental domain, CHV roles in antimicrobial waste management and community hygiene have been described by Musoke et al. [33] and Tsekleves et al. [42]. They can address the environmental AMR reservoir that increasingly drives community ESBL-E transmission in SSA [8].

The left panel of the framework identifies the key inputs and enablers that must be in place for CHV-led AMR programmes to function effectively: structured behaviour change training, access to point-of-care diagnostic tools, integration into existing community health platforms (particularly iCCM), robust supervision and mentorship systems, alignment with national AMR action plans, the foundational community trust that makes CHVs effective agents, and multi-sectoral partnerships that provide resources and technical support. The right panel catalogues the outcome domains that well-designed programmes can demonstrably achieve, as evidenced by the literature synthesised in this review. The bottom panel identifies the barrier modifiers that, if left unaddressed, will attenuate programme effectiveness at every node in the system, including resource constraints, training overload, unrestricted over-the-counter antibiotic access, regulatory and surveillance weaknesses, and persistent policy-to-practice gaps [10,4,47]. The framework also situates CHV AMR programmes within the broader structural environment: poverty, weak governance, and power asymmetries between communities and health systems are not merely peripheral contextual notes but central determinants of what CHV-led programmes can and cannot achieve [52,50]. These structural factors must be explicitly addressed in programme design and evaluation, not treated as fixed background conditions..

### Strengths and limitations

This review has several strengths. It is, to our knowledge, the first scoping review to comprehensively map CHV-led AMR interventions across SSA within an explicit One Health framework. The use of the PRISMA-ScR reporting standard enhances transparency and reproducibility. The inclusion of both peer-reviewed literature and grey literature is essential in a field where significant programmatic evidence exists outside traditional academic publication channels. The breadth of intervention types captured provides a genuinely comprehensive evidence landscape.

Limitations include the inherent heterogeneity of scoping reviews, which precludes formal meta-analytic synthesis. The language restriction to English and French may have excluded relevant evidence from Lusophone SSA countries. Publication and reporting bias may affect the peer-reviewed literature. As a scoping review, our findings map the landscape rather than providing definitive effectiveness estimates; a systematic review with meta-analysis would be a valuable next step as the evidence base matures. Furthermore, it should be explicitly acknowledged that whilst CHVs are well positioned to deliver human health-focused AMR interventions, they are generally less equipped, in terms of training, mandate, and resources, to address agricultural, environmental, and animal health dimensions of AMR. Realising the One Health potential of CHV programmes will therefore require substantially more integrated training, cross-sectoral collaboration, and dedicated resourcing than has been documented to date in SSA. The date restriction to January 2018 onwards, whilst justified by the post-UNGA 2016 AMR policy context, is acknowledged as a limitation that may have excluded earlier community-level evidence. Also, the recommendations herein are limited by the complex context of AMR interventions in LMICs, where Pokharel et al. [52] point out that the high burden of infectious diseases, extensive poverty levels, weak health systems, and low awareness of AMR continue to hinder the fight against AMR. There are also power differences, alignment of interests, poverty, and vulnerability, which may be systemically embedded and may not be amenable to CHW interventions [50].

### Future research priorities

Based on the evidence gaps identified in this review, we propose the following priority areas for future research: (1) Rigorous randomised or quasi-experimental trials with microbiological endpoints measuring the impact of CHV-led One Health AMR interventions on AMR outcomes in community settings; (2) Formal cost-effectiveness analyses of CHV AMR training and diagnostic integration programmes; (3) Evaluation of digital health platforms (mHealth, decision support algorithms) as components of CHV AMR capacity development; (4) Long-term (minimum 12-month) follow-up studies assessing the durability of CHV behaviour change; (5) Research explicitly targeting francophone SSA and other underrepresented regions; and (6) Comprehensive implementation science studies assessing scalability, fidelity, and adaptation of CHV AMR programmes across diverse SSA health system contexts. A seventh priority is qualitative and mixed-methods research exploring the structural, political, and ethical dimensions of CHV AMR programme implementation, including how power dynamics, poverty, and health system fragmentation shape what is achievable at the community level.

## Conclusion

CHVs represent a promising yet systematically neglected platform for AMR mitigation in SSA. This scoping review demonstrates that CHV-led interventions can generate meaningful improvements in antibiotic prescribing behaviour, AMR knowledge, infection prevention, and diagnostic stewardship. Their effectiveness is shaped by multiple macro- and micro-level determinants, and findings from individual contexts should not be uncritically generalised. CHVs are well positioned to address human health dimensions of AMR but remain less equipped to engage with agricultural, environmental, and animal health challenges without substantially more integrated training, cross-sectoral collaboration, and dedicated resourcing. The proposed conceptual framework offers a practical roadmap while explicitly acknowledging the structural constraints that bound this ambition. We call on governments, international partners, research funders, and health system architects to bring CHVs from the periphery to the centre of SSA’s AMR containment agenda, supported by the right enablers and evaluated against measurable outcomes.

## Supporting information

S1 Checklist PRISMA-ScR Checklist. Reproduced from Tricco et al. [23] under a CC BY 4.0 license.
(DOCX)

S1 Text Search strategy. (DOCX)
