# Supplementary material for: Community health volunteers as a frontline platform for antimicrobial resistance mitigation in sub-Saharan Africa: A scoping review
Source: PLOS Glob Public Health. 2026 Jun 11;6(6):e0006640. doi: 10.1371/journal.pgph.0006640 (PMC13258006; doi:10.1371/journal.pgph.0006640)
Supplement: S1 Text — (DOCX) [file pgph.0006640.s002.docx]

# Search Strategy

PubMed

*Filters applied: from 2018/1/1 - 2026/1/31.*

(community health volunteer*[tw] OR community health worker*[tw] OR CHW[tw] OR CHWs[tw] OR lay health worker*[tw] OR village health worker*[tw] OR community animal health worker*[tw] OR health extension worker*[tw] OR community health aide*[tw] OR "Community Health Workers"[Mesh] OR community health[tw] OR health volunteer*[tw] OR volunteer health worker*[tw] OR frontline health worker*[tw] OR grassroots health worker*[tw] OR promotora*[tw] OR promotor*[tw] OR barefoot doctor*[tw] OR village health agent*[tw] OR community based health worker*[tw] OR community health promoter*[tw] OR health surveillance assistant*[tw] OR health extension agent*[tw] OR community health officer*[tw] OR village health team*[tw] OR community case manager*[tw] OR integrated community case manager*[tw] OR ICCM[tw] OR community drug distributor*[tw] OR community health volunteer[tw] OR village health helper[tw] OR community health liaison[tw] OR community health representative[tw] OR family health worker*[tw] OR community health navigator*[tw]) AND ("Drug Resistance, Microbial"[Mesh] OR antimicrobial resistance[tw] OR AMR[tw] OR antibiotic resistance[tw] OR antimicrobial stewardship[tw] OR antibiotic stewardship[tw] OR AMS[tw] OR drug resistance[tw] OR antibiotic use[tw] OR antibiotic prescribing[tw] OR antibiotic prescription*[tw] OR rational use[tw] OR rational drug use[tw] OR infection prevention[tw] OR infection control[tw] OR IPC[tw] OR antibiotic misuse[tw] OR antimicrobial misuse[tw] OR drug resistant[tw] OR drug-resistant[tw] OR ESBL[tw] OR ESBL-producing[tw] OR extended spectrum beta lactamase[tw] OR multidrug resistant[tw] OR multi-drug resistant[tw] OR MDR[tw] OR extensively drug resistant[tw] OR XDR[tw] OR pan-drug resistant[tw] OR PDR[tw] OR carbapenem resistant[tw] OR colistin resistant[tw] OR methicillin resistant[tw] OR MRSA[tw] OR vancomycin resistant[tw] OR VRE[tw] OR antimicrobial prescribing[tw] OR inappropriate antibiotic*[tw] OR overuse[tw] OR overuse of antibiotic*[tw] OR antibiotic consumption[tw] OR antimicrobial consumption[tw] OR stewardship[tw] OR antibiotic resistance genes[tw] OR resistome[tw] OR one health[tw] OR WASH[tw] OR hygiene[tw] OR sanitation[tw] OR water quality[tw] OR wastewater[tw] OR environmental contamination[tw] OR veterinary antibiotic*[tw] OR livestock antibiotic*[tw] OR agricultural antibiotic*[tw] OR antimicrobial dispensing[tw] OR antibiotic dispensing[tw] OR antibiotic access[tw] OR antibiotic availability[tw] OR non-prescription antibiotic*[tw] OR over the counter antibiotic*[tw] OR OTC antibiotic*[tw] OR informal antibiotic*[tw] OR leftover antibiotic*[tw] OR antibiotic sharing[tw] OR incomplete course[tw] OR treatment abandonment[tw] OR diagnostic stewardship[tw] OR point of care testing[tw] OR POC testing[tw] OR C-reactive protein[tw] OR CRP testing[tw] OR rapid diagnostic test*[tw] OR RDT[tw]) AND ("Africa South of the Sahara"[Mesh] OR sub-Saharan Africa[tw] OR sub Saharan Africa[tw] OR Africa[tw] OR Uganda[tw] OR Kenya[tw] OR Tanzania[tw] OR Zambia[tw] OR Malawi[tw] OR Ethiopia[tw] OR Ghana[tw] OR Nigeria[tw] OR Zimbabwe[tw] OR Sierra Leone[tw] OR Burkina Faso[tw] OR Mozambique[tw] OR Rwanda[tw] OR Senegal[tw] OR Cameroon[tw] OR South Africa[tw] OR Angola[tw] OR Benin[tw] OR Botswana[tw] OR Burundi[tw] OR Cabo Verde[tw] OR Cape Verde[tw] OR Central African Republic[tw] OR Chad[tw] OR Comoros[tw] OR Congo[tw] OR Democratic Republic of Congo[tw] OR Cote d'Ivoire[tw] OR Ivory Coast[tw] OR Djibouti[tw] OR Equatorial Guinea[tw] OR Eritrea[tw] OR Eswatini[tw] OR Swaziland[tw] OR Gabon[tw] OR Gambia[tw] OR Guinea[tw] OR Guinea-Bissau[tw] OR Lesotho[tw] OR Liberia[tw] OR Madagascar[tw] OR Mali[tw] OR Mauritania[tw] OR Mauritius[tw] OR Namibia[tw] OR Niger[tw] OR Sao Tome[tw] OR Somalia[tw] OR South Sudan[tw] OR Sudan[tw] OR Togo[tw] OR West Africa[tw] OR East Africa[tw] OR Central Africa[tw] OR Southern Africa[tw] OR Sahel[tw] OR Africa South of the Sahara[tw] OR SSA[tw])

Equivalent searches were adapted and executed in

1. EMBASE (via Ovid)
2. Cochrane Library (Cochrane Database of Systematic Reviews and CENTRAL)
3. AJOL.

Grey literature searches were conducted in:

1. WHO IRIS (keywords: community health volunteers AND antimicrobial resistance)
2. ReAct Africa website
3. AMREF document repository
4. UNICEF programme library
